# GPR68 deletion impairs hippocampal long-term potentiation and passive avoidance behavior

**DOI:** 10.1186/s13041-020-00672-8

**Published:** 2020-09-29

**Authors:** Yuanyuan Xu, Mike T. Lin, Xiang-ming Zha

**Affiliations:** Department of Physiology and Cell Biology, University of South Alabama College of Medicine, 5851 USA Dr. N, MSB3074, Mobile, AL 36688 USA

**Keywords:** OGR1, Synaptic plasticity, Fear memory

## Abstract

Increased neural activities reduced pH at the synaptic cleft and interstitial spaces. Recent studies have shown that protons function as a neurotransmitter. However, it remains unclear whether protons signal through a metabotropic receptor to regulate synaptic function. Here, we showed that GPR68, a proton-sensitive GPCR, exhibited wide expression in the hippocampus, with higher expression observed in CA3 pyramidal neurons and dentate granule cells. In organotypic hippocampal slice neurons, ectopically expressed GPR68-GFP was present in dendrites, dendritic spines, and axons. Recordings in hippocampal slices isolated from GPR68−/− mice showed a reduced fiber volley at the Schaffer collateral-CA1 synapses, a reduced long-term potentiation (LTP), but unaltered paired-pulse ratio. In a step-through passive avoidance test, GPR68−/− mice exhibited reduced avoidance to the dark chamber. These findings showed that GPR68 contributes to hippocampal LTP and aversive fear memory.

Brain acidification occurs in both physiological and disease conditions. A better understanding of how protons regulate synaptic physiology will help advancing our knowledge of brain physiology and pathophysiology. However, it remains unclear whether protons signal through metabotropic receptors to alter synaptic function. GPR68 (also known as OGR1-ovarian cancer G protein-coupled receptor 1) is a proton-sensitive GPCR expressed in brain neurons. GPR68 starts to get activated at about pH 7.4, reaches maximal activation at ~ 6.8–6.5, and primarily couples to Gq_/11_ to elicit intracellular phospholipase C/calcium signaling [1–3]. Several histidine residues are important for its proton sensing [2]. Potentiating GPR68 function in mice alters fear memory [4]. These studies suggest a potential role of GPR68 in synaptic function.

To determine whether GPR68 contributes to synaptic physiology, we started by examining the expression of GPR68 in hippocampus. Since there are no reliable antibodies to detect endogenous GPR68 (as controlled by GPR68−/− tissue, not shown), we utilized a Tg(*Gpr68*-eGFP) mouse line which expresses GFP under the control of *Gpr68* promoter [5]. Thus, in this transgenic mouse, expression pattern of GFP reflects that of GPR68 in vivo. We performed cryosections of brains isolated from wild-type (WT, negative control for GFP staining) and the Tg(*Gpr68*-eGFP) mice, and stained the sections using a GFP antibody (see Additional File 1 for detailed methods for all experiments). Within the hippocampus,  dentate granule cells and CA3 pyramidal neurons exhibited higher expression while CA1 pyramidal neurons exhibited lower staining (Fig. 1a). This expression pattern is consistent with the ISH result of Allen Brain Atlas. In addition, we observed diffuse GFP signals throughout stratum radiatum and stratum oriens (Fig. 1a). To gain more information on subcellular distribution of GPR68 in neurons, we transfected organotypic hippocampal slices biolistically [6] with a construct encoding a GPR68-eGFP fusion protein together with a membrane targeted Lck-mStrawberry, which marks transfected cell. Biolistic transfection only targets a few neurons, thus allows greatly improved signal-to-noise ratio in immunofluorescence imaging of the transfected protein. We performed immunostaining for GFP and visualized the distribution of GPR68-GFP with confocal microscopy. GPR68 was present throughout dendritic branches, axons, and the majority of dendritic spines (Fig. 1b).

**Fig. 1:**
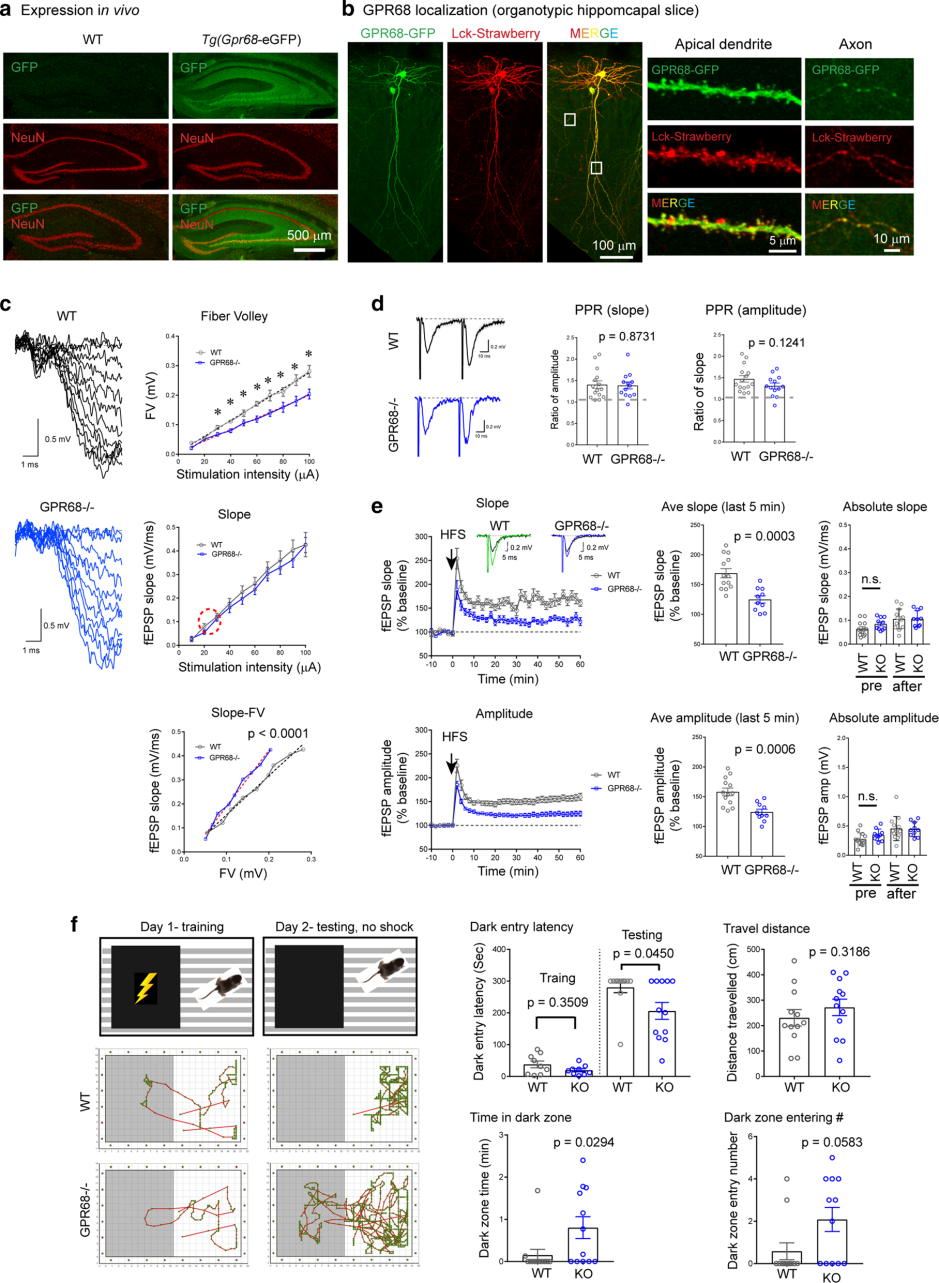
**a** Confocal images showing GFP (in green) and NeuN (in red) immunofluorescence in hippocampus of WT (negative control) and Tg(*Gpr68*-eGFP) mice. The Tg(*Gpr68*-eGFP) mouse expresses eGFP under the control of Gpr68 promoter. **b** Localization of GPR68-GFP fusion protein in organotypic hippocampal slices. Organotypic hippocampal slices were biolistically transfected with GPR68-GFP together with Lck-mStrawberry, which serves as a marker for transfected cells. To reveal relatively weak GFP68-GFP signals, GFP immunofluorescence was performed using an anti-GFP antibody. Leftmost set of images show an overall view while the right two sets show high-magnification images of a segment of apical dendrite and axon of a transfected CA1 neuron (boxed regions on the left). **c** Input/Output responses. Traces on the left are representative for input/output recordings from WT and GPR68−/− slices. Graphs on the right show the quantification of FV, fEPSP slope, and fEPSP slop-FV relationship. The red circle marks the approximate range of stimulation/response used in the LTP study (see panel E, plot of Absolute Slope). * Denotes statistical significance (*p* < 0.05, 2-tailed t-test; n = 8 WT and 12 GPR68−/− slices). p value for slope-FV relationship was obtained from linear regression analysis comparing the slopes (dashed lines) of the two genotypes. **d** Paired-pulse facilitation. Representative traces (left panel) and quantification of paired-pulse ratio for slope (middle panel) and amplitude (right panel). The two stimulations were evoked at 50 ms interval. Each dot represents one hippocampal slice. p values were obtained from 2-tailed t-tests. **e** Hippocampal LTP. Changes in slope (top panel) and amplitude (bottom panel) of fEPSP in the CA1 region before and after high frequency stimulation (HFS: 100 Hz 1 s). Insets in the top plot show representative averages of 10 fEPSP traces before and 1 h after HFS for WT and GPR68−/−. Summary graphs in the middle were averages of the last 5 min of recordings. Graphs on the right show the absolute slope and amplitude, which was average for 3 min before (pre) and at the end of LTP (57–60 min). Note that the baseline (pre) and post-LTP slopes were at 20–30% of the maximum slope shown in **c**. The p values for baseline comparisons were 0.102 (for slope) and 0.0981 (for amplitude). Each dot represents one hippocampal slice. p values were obtained from 2-tailed t-tests. **f** Passive avoidance test. Diagrams show the training and test scheme (upper panel). Traces below show typical movement traces of a WT (middle panel) and a knockout (lower panel) mice during training and testing sessions. Quantifications show latency to enter the dark chamber on the training and test days, total travel distance, time spent in the dark chamber, and number of times entering the dark chamber during the 5 min recording on the test day. p values were obtained from 2-tailed Mann–Whitney U test. Each dot represents one male animal

To determine whether GPR68 contributes to synaptic transmission, we first assessed input/output responses at the Schaffer collateral-CA1 synapse of WT and GPR68−/− [7] slices. Increasing stimulus intensities (0–100 μA) resulted in an increase in field EPSP (fEPSP) in both genotypes (Fig. 1c). To determine whether the presynaptic response was different, we quantified the fiber volley (FV). GPR68 deletion significantly reduced FV. However, the postsynaptic fEPSP slope did not differ between WT and GPR68−/−. It is possible that the altered FV kinetics do not influence postsynaptic responses in the knockout. We further plotted the fEPSP slope-FV relationship in WT and GPR68−/− slices. The fitted slope for WT was 1.629 ± 0.072, significantly lower than that of GPR68−/− (2.327 ± 0.099; p < 0.0001, linear regression analysis). Mechanisms for this phenomenon remain unclear. The abundant GPR68 expression in CA3 neurons suggests a potential presynaptic site of action. Other possibilities include an increase in quantal size or the number of release sites, and/or postsynaptic compensation.

Next, we investigated the ratio of paired-pulse facilitation, which reflects changes in presynaptic release efficacy. Paired-pulse stimulation, separated by 50 ms intervals, induced comparable increases in the size of the second fEPSP in WT and GPR68−/− slices (Fig. 1d). To determine whether GPR68 deletion alters hippocampal LTP, we performed fEPSP recording before and after a train of high frequency stimulation (HFS, 100 Hz for 1 s). In WT slices, HFS induced a persistent increase in fEPSP (Fig. 1e). At 55–60 min following HFS, the averaged fEPSP for WT slices was 169 ± 7.7% (slope) and 158 ± 6.4% (amplitude). Deleting GPR68 attenuated the magnitude of LTP. In GPR68−/− slices, both the slope (125 ± 6.1%) and amplitude (124 ± 4.8%) were significantly (p = 0.0003 for slope and 0.0006 for amplitude, 2-tailed t-test) lower as compared to WT. To better compare fEPSP values before normalization, we further plotted the absolute values of fEPSP slope and amplitude (3-min averages) before HFS and at the end of LTP recording. For both genotypes, the averaged responses were around 20–30% of the maximal response (compare with the slope-stimulation plot of panel c, middle graph). There were no significant differences between the two genotypes in baseline slope or amplitude.

To determine whether the knockout exhibits learning deficits, we performed a step-through passive avoidance test. This test is based on the innate aversion of rodents to brightly illuminated areas and on their exploratory behavior to novel environment [8]. On day-1 (training), we placed the mouse in a brightly illuminated chamber (Fig. 1f). When the mouse entered the dark chamber, the instrument delivered a single footshock. On day-2, we returned the mouse to the same chamber and recorded its movement for 5 min. Both genotypes exhibited avoidance to the dark chamber, as evidenced by their travel pattern and latency to enter (Fig. 1f). Compared to the WT, the knockout exhibited significant reduction in dark entry latency, increase in dark chamber presence, and a trend of an increase in the number of times entering the dark chamber. The deficits in avoidance memory is consistent with a reduced hippocampal LTP. However, other brain regions may also contribute to behavioral changes in GPR68−/−.

Of note, a previous study examined a different GPR68−/− mouse line and reported no differences in context- or cued-fear using a fear conditioning paradigm which applied 3 trains of 2-s footshocks during training [4]. Here, we used a passive avoidance test. In our training, we delivered a single 1-s footshock, which was sufficient to elicit robust avoidance response (comparing Training vs. Testing) and yet revealed a difference between the two genotypes. Of note, in our preliminary trial to determine the optimal protocol, a single 2-s footshock elicited robust avoidance and greatly reduced movement (“freezing”) in a small cohort (2 WT and 1 KO) of animals: none of these animals enter the dark chamber during the testing; the average travel distance was 107.3 ± 37.4 cm, less than 50% of that following the 1-s protocol. We did not pursue this protocol further because this preliminary result, though with very small N, suggested that the 2-s shock in our testing was too strong. Nevertheless, it will be of future interest to determine whether an increased shocking paradigm occludes the effect of GPR68 in various learning paradigms.

In summary, our results here showed that GPR68 was present in hippocampal pyramidal neurons with higher expression in CA3 and dentate gyrus. GPR68 deletion reduced FV at the Schaffer collateral-CA1 synapse, attenuated hippocampal LTP, and led to deficits in the step-through passive avoidance test. How GPR68 contributes to LTP warrants further investigation. Synaptic activities can reduce cleft pH by a few tenths of pH units [9–11]. This pH reduction is sufficient to activate GPR68, which reaches maximal activation at pH 6.8–6.5 [1, 12]. GPR68 activates phospholipase C/calcium signaling [1–3]. It will be of future interest to determine whether any of these effectors mediate the synaptic effect of GPR68. The findings on GPR68 provide a novel pathway, independent of the previously described ASIC-dependent pathways [10, 13–15], to explain the synaptic effect of protons. Since persistent acidosis is prevalent in disease conditions while GPR68 do not exhibit rapid desensitization [1, 12], GPR68-dependent mechanisms arguably would have close relevance to long-term psychophysiological changes commonly seen in various neurological diseases.

## Supplementary information

**Additional file 1.** Supplemental information containing detailed Material and Methods and Supplemental References.

## Data Availability

All data generated or analyzed during this study are included in this published article. Additional inquiries can be directed to the corresponding author.
