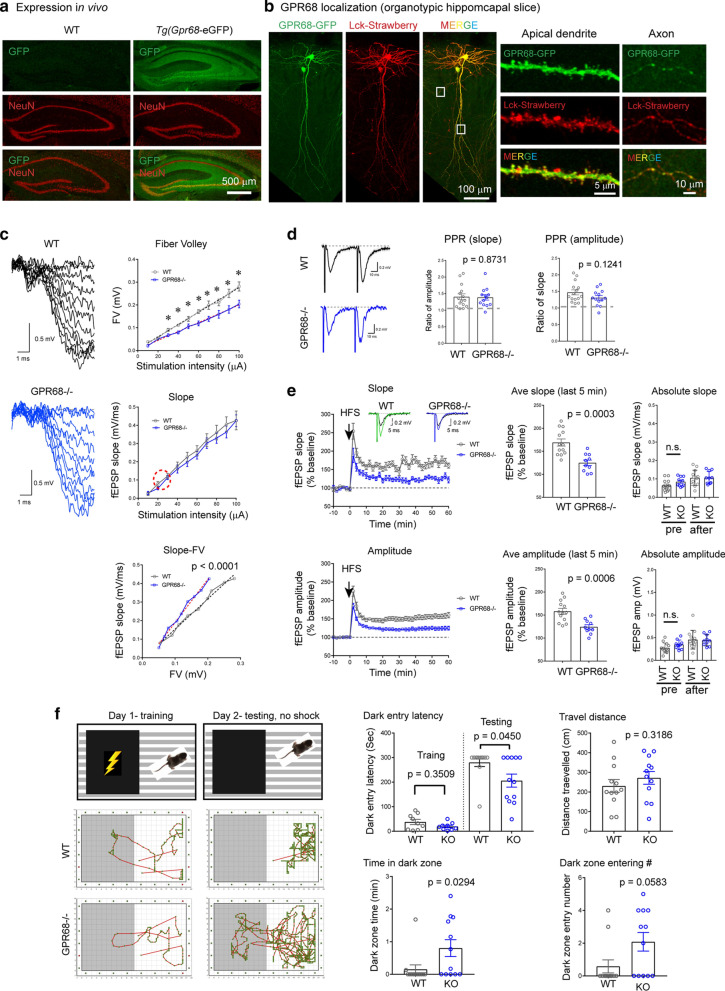# Correction to: GPR68 deletion impairs hippocampal long-term potentiation and passive avoidance behavior

**DOI:** 10.1186/s13041-021-00742-5

**Published:** 2021-02-23

**Authors:** Yuanyuan Xu, Mike T. Lin, Xiang-ming Zha

**Affiliations:** Department of Physiology and Cell Biology, University of South Alabama College of Medicine, 5851 USA Dr. N, MSB3074, Mobile, AL 36688 USA

## Correction to: Mol Brain (2020) 13:132 https://doi.org/10.1186/s13041-020-00672-8

Following publication of the original article [1], the authors identified an error in Fig. 1. In Fig. 1a, the two NeuN images (2nd row) for WT and tg(Gpr68) were reversed. The incorrect and correct figure are published in this Correction article. The original article has been updated.

Correct Figure 1 
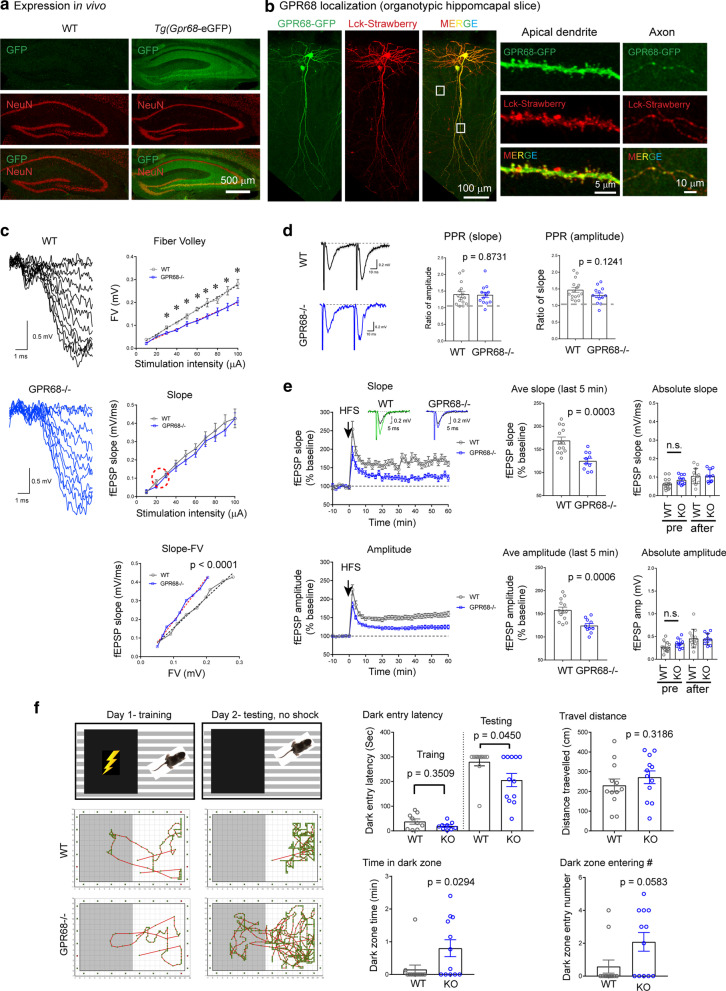
**Fig. 1** **a** Confocal images showing GFP (in green) and NeuN (in red) immunofluorescence in hippocampus of WT (negative control) and Tg(*Gpr68*-eGFP) mice. The Tg(*Gpr68*-eGFP) mouse expresses eGFP under the control of Gpr68 promoter. **b** Localization of GPR68-GFP fusion protein in organotypic hippocampal slices. Organotypic hippocampal slices were biolistically transfected with GPR68-GFP together with Lck-mStrawberry, which serves as a marker for transfected cells. To reveal relatively weak GFP68-GFP signals, GFP immunofluorescence was performed using an anti-GFP antibody. Leftmost set of images show an overall view while the right two sets show high-magnification images of a segment of apical dendrite and axon of a transfected CA1 neuron (boxed regions on the left). **c** Input/Output responses. Traces on the left are representative for input/output recordings from WT and GPR68−/− slices. Graphs on the right show the quantification of FV, fEPSP slope, and fEPSP slop-FV relationship. The red circle marks the approximate range of stimulation/response used in the LTP study (see panel E, plot of Absolute Slope). * Denotes statistical significance (*p* < 0.05, 2-tailed t-test; n = 8 WT and 12 GPR68−/− slices). p value for slope-FV relationship was obtained from linear regression analysis comparing the slopes (dashed lines) of the two genotypes. **d** Paired-pulse facilitation. Representative traces (left panel) and quantification of paired-pulse ratio for slope (middle panel) and amplitude (right panel). The two stimulations were evoked at 50 ms interval. Each dot represents one hippocampal slice. p values were obtained from 2-tailed t-tests. **e** Hippocampal LTP. Changes in slope (top panel) and amplitude (bottom panel) of fEPSP in the CA1 region before and after high frequency stimulation (HFS: 100 Hz 1 s). Insets in the top plot show representative averages of 10 fEPSP traces before and 1 h after HFS for WT and GPR68−/−. Summary graphs in the middle were averages of the last 5 min of recordings. Graphs on the right show the absolute slope and amplitude, which was average for 3 min before (pre) and at the end of LTP (57–60 min). Note that the baseline (pre) and post-LTP slopes were at 20–30% of the maximum slope shown in **c**. The p values for baseline comparisons were 0.102 (for slope) and 0.0981 (for amplitude). Each dot represents one hippocampal slice. p values were obtained from 2-tailed t-tests. **f** Passive avoidance test. Diagrams show the training and test scheme (upper panel). Traces below show typical movement traces of a WT (middle panel) and a knockout (lower panel) mice during training and testing sessions. Quantifications show latency to enter the dark chamber on the training and test days, total travel distance, time spent in the dark chamber, and number of times entering the dark chamber during the 5 min recording on the test day. p values were obtained from 2-tailed Mann–Whitney U test. Each dot represents one male animal

Incorrect Figure 1