# Supplementary material for: GPR68 deletion impairs hippocampal long-term potentiation and passive avoidance behavior
Source: Mol Brain. 2020 Sep 29;13:132. doi: 10.1186/s13041-020-00672-8 (PMC7526169; doi:10.1186/s13041-020-00672-8)
Supplement: Supplementary file 1 — Additional file 1. Supplemental information containing detailed Material and Methods and Supplemental References. [file 13041_2020_672_MOESM1_ESM.pdf]

ADDITIONAL FILE 1

GPR68 deletion impairs hippocampal long-term potentiation and passive avoidance behavior.

Yuanyuan Xu, Mike T. Lin, and Xiang-ming Zha*

SUPPLEMENTAL INFORMATION

MATERIAL and METHODS

Mice

GPR68-/- mice were generated previously [1] and maintained on a congenic C57BL/6 background. The Tg(*Gpr68*-eGFP) reporter mouse line was purchased from MMRRC. To minimize the drift of genetic background, the knockouts were backcrossed to wild-type C57BL/6 every 6-10 generations, following the recommendations of the Jackson Laboratory. Wild-type (WT) and GPR68-/- mice were generated from breeding of heterozygous littermates, and maintained as breeding colonies at the University of South Alabama. The Tg(*Gpr68*-eGFP) reporter mouse used in this study was on a mixed CD1:C57 background. All procedures were approved by the University of South Alabama Animal Care and Use Committee.

Constructs and Reagents

Constructs encoding GPR68-eGFP and Lck-mStrawberry was generated by PCR mediated subcloning. All constructs contained the same Kozak sequence GCCACCATG and were verified by sequencing. Anti-GFP antibody used include a mouse monoclonal (Neuromab 75-131) or a rat monoclonal (Nacalai GF090R) antibody. Medium and serum for tissue culture were purchased from ThermoFisher.

Brain isolation, Cryosection, and Immunostaining

The mice were perfused with phosphate buffered saline (PBS) or saline, followed by 30-50 ml of 4% paraformaldehyde in PBS. The brains were then isolated and post-fixation in 4% paraformaldehyde (for one night), protected overnight in 15% and then 25 or 30% sucrose, and sectioned coronally at 12 or 16 µm thickness. For GFP staining in GPR68-GFP reporter line, 2-12 month old mice were used. For immuostaining, we used a protocol similar as previously described [2]. Briefly, the slices were rehydrated and permeabilized with PBS 1% Triton for 2 hr. For GFP staining, a pre-treatment step with 1% SDS for 60 sec was adopted to reduce non-specific signals. The sections were blocked sequentially (20-30 min each at room temperature) with 50 mM NH_4Cl and blocking buffer (PBS, 10% Horse serum, 5 mg/ml BSA, 0.2% Triton X-100). For antibody dilution and washing, the buffer was a 1:4 dilution of the blocking buffer with PBS. Primary antibodies were incubated overnight at 4°C in a moisturized dark box, washed three times (5-10 min per wash with gentle rocking at room temperature) with washing buffer, and added Alexa- or DyLight-conjugated secondary antibodies (1:600-1000 dilutions) and incubated at room temperature for 2 hr. Slides were mounted with either a homemade 50% glycerol based mounting media or Permount (EMS), and imaged with a Nikon A1R confocal microscope.

Organotypic Hippocampal Slice culture, Transfection, and Immunofluorescence

Organotypic mouse hippocampal slices were cultured as described earlier [3, 4]. Briefly, transverse sections (350 µm thick) were cut with a tissue chopper. The slices were then transferred onto Falcon polyethylene terephthalate-etched membrane culture inserts with 1 µm pores (Fisher) in 6 well plates. Culture medium was: 25% HBSS, 25% horse serum and

50% MEM with 2 mM GlutaMax, 1.5 mg/ml glucose, 5U/ml pen/strep and supplemented with extra 4.5 mM $NaHCO_3$. Slice cultures were kept in a humidified CO_2 incubator maintained at 37°C 5.5% CO_2 and fed every 2-3 days. Transfection was performed after 7-10 days in culture with a Helios gene gun (Bio-Rad) at 70-75 psi, similar to what was described earlier [3, 4]. Two days after transfection, slices were fixed with 4% paraformaldehyde in Hanks' balanced salt solution with calcium and magnesium containing 6 mg/mL glucose and 20 mM HEPES (pH 7.3) for 10-15 min, washed 3 times with PBS with calcium and magnesium. Immunofluorescence was performed as described previously [4].

Confocal Microscopy

Confocal images were captured using a laser scanning microscope (Nikon A1), similar to that described earlier [4]. Briefly, illumination was provided by an argon (Ar, 458, 488, 514 nm lines) and a 561 nm diode laser. Green and red channels were imaged sequentially to eliminate bleed-through, using 488 nm excitation and a 525/50 emission filter and 561 nm excitation and a 595/50 emission filter, respectively. Images were captured with a 20x/0.75 multi-immersion lens or a PL APO 63x/1.2 water lens. To visualize spines, a series of high-resolution images (1024 x 256 to 1024 x 1024 pixel array) were captured at a z-step of 0.4- 0.5 μm with an additional electronic zoom of 3-4. Each plane was imaged with an average of 4 scans. Maximal projection images were exported and presented.

Acute Hippocampal Slice Preparation and Electrophysiological Recordings

Hippocampal slices were prepared from 7–9-wk-old Male mice, similar to earlier descriptions [5]. Briefly, mice were anesthetized with isoflurane and rapidly decapitated. The brain was quickly removed and placed in ice-cold sucrose-containing artificial cerebrospinal fluid (ACSF) (in millimolar: 70 sucrose, 80 NaCl, 2.5 KCl, 21.4 $NaHCO_3$, 1.25 NaH_2PO_4, 0.5 $CaCl_2$, 7 $MgCl_2$, 1.3 ascorbic acid, and 20 glucose). Transverse hippocampal slices (350 μm) were cut with slice chopper and transferred into a holding chamber containing recording ACSF (in millimolar: 125 NaCl, 2.5 KCl, 21.5 $NaHCO_3$, 1.25 NaH_2PO_4, 2.0 $CaCl_2$, 1.0 $MgCl_2$, and 15 glucose). Slices were incubated at 35°C for 30 min and then at room temperature for 1 hr before recordings were performed. All solutions were constantly equilibrated with 95% O_2 and 5% CO_2.

Electrophysiological recording of field EPSP was performed at room temperature, with the recording chamber continuously superfused with ACSF at the rate of 1.5 ml/min. Capillary glass pipettes were filled with ACSF and had tip resistance of ~1 MΩ. A stimulation electrode was placed in stratum radiatum ~100 μm from the CA1 pyramidal cell body, and ~150 μm from the dendrite. The stimulus duration of each pulse was 0.1 ms which allowed for clear separation of fiber volley (FV) from the preceding stimulus artifact and the postsynaptic response. Recordings were obtained using an EPC 10 amplifier (Heka, Lambrecht (Pfalz), Germany). Analog signals were further amplified 10 times and filtered at 5 kHz using an Axopatch amplifier (Molecular Devices, San Jose, CA, USA) and digitized at 20 kHz using Patchmaster software (Heka). Synaptic input/output and paired-pulse ratio were recorded prior to LTP recording. For LTP, baseline responses were collected with a stimulation intensity that yielded 20-30% of the maximal responses before the appearance of population spike. After obtaining a stable baseline for 10-20 min, LTP was induced by a train of high-frequency stimulation (HFS: 100 Hz for 1 sec). The field excitatory postsynaptic potential (EPSP) was recorded for another 60 min after HFS.

Light/dark preference test and passive avoidance test

For all behavioral assessment, the animals were transferred to the behavioral room and let accustomed for at least 20 min before the testing. WT and GPR68-/- male animals were

matched in age on training and test performed on the same day. For step-through passive avoidance test, a dark colored box is put in a brightly illuminated cage inside an infrared SmartCage system (AfaSci). The dark box was set on a metal grid within the SmartCage. The mouse was initially placed outside of the dark box with its head facing away from the dark box. The mouse can freely move inside and outside of the dark box through a door at the bottom of the dark box. When the mouse entered the dark box, a single foot shock (68-70 V) of 1 second was delivered. Immediately following the shock, the mouse was removed from the SmartCage and returned to its home cage. Its movement was recorded and saved by SmartCage. At about 24 hr after the training, the mouse was returned to the test cage with the dark box on metal grid. The mouse was placed in the light chamber and its activity was monitored for 5 min. No foot shock was delivered on day-2 whether or not the mouse was in the dark box. Due to an error, the training traces were not recorded for 3 WT and 3 GPR68-/-. As a result, the N number for dark entry latency during training were 9 WT and 9 GPR68-/-, 3 less for each genotype than those (12 WT, 12 GPR68-/-) for testing (see Figure 1F). Raw data were exported from AfaSci and further analyzed and plotted in Microsoft Excel and/or GraphPad.

Data Analysis and Statistics

Electrophysiology data were analyzed using Igor Pro (WaveMetrics) and exported to Microsoft Excel or Graphpad for further analysis. For LTP plot, binning data at 2 min intervals generated summary plots. For comparing of changes at the end of LTP, average amplitude or slope % baseline was quantified at 55-60 min of recording. Behavioral analysis was performed in Excel and GraphPad. Data are expressed as mean ± s.e.m. Two-tailed t-test or Mann-Whitney U test were used to determine significance of data between WT and GPR68-/- groups. For field EPSP slope-fiber volley relationship (Figure 1C), linear regression analysis was used to compare the slope of the two curves. $p < 0.05$ was considered significant.

Supplemental References:

1. Li H, Wang D, Singh LS, Berk M, Tan H, Zhao Z, Steinmetz R, Kirmani K, Wei G, Xu Y: Abnormalities in osteoclastogenesis and decreased tumorigenesis in mice deficient for ovarian cancer G protein-coupled receptor 1. *PLoS One* 2009, 4(5):e5705.
2. Jiang N, Wu J, Leng T, Yang T, Zhou Y, Jiang Q, Wang B, Hu Y, Ji YH, Simon RP *et al*: Region specific contribution of ASIC2 to acidosis-and ischemia-induced neuronal injury. *J Cereb Blood Flow Metab* 2017, 37(2):528-540.
3. Zha XM, Wemmie JA, Green SH, Welsh MJ: Acid-sensing ion channel 1a is a postsynaptic proton receptor that affects the density of dendritic spines. *Proc Natl Acad Sci U S A* 2006, 103(44):16556-16561.
4. Jing L, Chu XP, Jiang YQ, Collier DM, Wang B, Jiang Q, Snyder PM, Zha XM: N-glycosylation of acid-sensing ion channel 1a regulates its trafficking and acidosis-induced spine remodeling. *J Neurosci* 2012, 32(12):4080-4091.
5. Balczon R, Pittet JF, Wagener BM, Moser SA, Voth S, Vorhees CV, Williams MT, Bridges JP, Alvarez DF, Koloteva A *et al*: Infection-induced endothelial amyloids impair memory. *FASEB J* 2019, 33(9):10300-10314.
